# Molecular Analysis of Trypanosome Infections in Algerian Camels

**DOI:** 10.1007/s11686-022-00577-7

**Published:** 2022-06-03

**Authors:** Djamila Boushaki, Julie Wallis, Frederik Van den Broeck, Achim Schnaufer

**Affiliations:** 1 Inspection Vétérinaire de la Wilaya d’Alger, Direction des Services Agricoles, Ministère de l’Agriculture et du Développement Rural, Alger, Algeria; 2 Ecole Nationale Supérieure Vétérinaire, Alger, Algeria; 3 Institute of Immunology and Infection Research, University of Edinburgh, Edinburgh, UK; 4 Biomedical Sciences Department, Institute of Tropical Medicine, Antwerp, Belgium; 5 Department of Microbiology, Immunology and Transplantation, Rega Institute for Medical Research, Katholieke Universiteit Leuven, Leuven, Belgium

**Keywords:** Surra, *Trypanosoma brucei evansi*, kDNA, Kinetoplast, Minicircle

## Abstract

**Purpose:**

Surra is an economically important livestock disease in many low- and middle-income countries, including those of Northern Africa. The disease is caused by the biting fly-transmitted subspecies *Trypanosoma brucei evansi*, which is very closely related to the tsetse-transmitted subspecies *T. b. brucei* and the sexually transmitted subspecies *T. b. equiperdum*. At least two phylogenetically distinct groups of *T. b. evansi* can be distinguished, called type A and type B. These evolved from *T. b. brucei* independently. The close relationships between the *T. brucei* subspecies and the multiple evolutionary origins of *T. b. evansi* pose diagnostic challenges.

**Methods:**

Here we use previously established and newly developed PCR assays based on nuclear and mitochondrial genetic markers to type the causative agent of recent trypanosome infections of camels in Southern Algeria.

**Results/conclusion:**

We confirm that these infections have been caused by *T. b. evansi* type A. We also report a newly designed PCR assay specific for *T. b. evansi* type A that we expect will be of diagnostic use for the community.

**Supplementary Information:**

The online version contains supplementary material available at 10.1007/s11686-022-00577-7.

## Introduction

The single cellular parasite *Trypanosoma brucei evansi* belongs to the subgenus *Trypanozoon* that also comprises *T. b. brucei* and *T. b. equiperdum* [1, 2] (the taxonomical status of *T. b. evansi* is controversial [1, 3, 4]; for this study, we will be referring to it as a subspecies of *T. brucei*). *Trypanosoma b. evansi* is the most widely distributed of the pathogenic animal trypanosomes, affecting a large number of wild and domesticated animal species in Asia, Africa and Latin America [5, 6]. In Europe, it is present in the Canary Islands, from where recent sporadic incursions into the French and Spanish mainland have occurred [5, 7, 8]. *Trypanosoma b. evansi* causes a trypanosomosis called “surra” in many countries [8–10]. It is an acute, chronic or subclinical disease that is very often fatal in camels, horses and dogs, but can also seriously affect cattle and buffaloes. Other animals, including wildlife, are also susceptible.

In affected countries, surra is an economically important disease, which causes high mortality, reduced milk and meat production, poor carcass quality, reduced reproductive performance, and decreased draft power and manure production [9]. Haematophagous flies of the genera *Tabanus* and *Stomoxys* are particularly relevant for transmitting the infection from host to host, acting as mechanical vectors without parasite development in the insect [9]. This is a key difference to *T. b. brucei*, where transmission is dependent on cyclic development in the tsetse fly [11]. Indeed, it is this mechanical transmission that has allowed the parasite to move beyond the tsetse fly region and out of Africa [12]. In South and Central America, *T. b. evansi* can also be transmitted by vampire bats (*Desmodus rotundus*), which act as both vectors and reservoirs [9].

Another key difference to *T. b. brucei* is that *T. b. evansi* strains are either ‘dyskinetoplastic’ or ‘akinetoplastic’, i.e., they have either dysfunctional kinetoplast DNA (the mitochondrial DNA network in these organisms) or lack it entirely. Where kDNA is present, *T. b. evansi* strains typically lack maxicircles—the equivalent of mitochondrial DNA in other eukaryotes—and are characterized by minicircle sequence homogeneity [13, 14]. By contrast, *T. b. brucei* contains hundreds of different minicircle classes [15]. *Trypanosoma b. evansi* is therefore incapable of mitochondrial gene expression, and a compensatory mutation in the nuclearly encoded subunit γ of the F_1_F_O_ ATP synthase is necessary to enable viability [16]. Based on the minicircle class that dominates the kDNA networks, *T. b. evansi* can be divided into types A and B [1, 13, 17]. Indeed, this difference can be exploited for polymerase chain reaction (PCR)-based diagnostics and molecular characterization of the parasite. PCR-based assays that target *Trypanozoon*-specific satellite DNA or ribosomal DNA are regarded as the most sensitive for diagnosis or characterization of surra infections [18–20], while for genotyping *T. b. evansi* and/or to distinguish between *T. b. evansi* types A and B, PCR assays targeting type-specific variant surface glycoprotein genes, mitochondrial minicircles and maxicircles, microsatellite markers and the F_1_-ATP synthase γ subunit gene are being used [4, 17, 21–23].

In northern Africa, the first cases of trypanosomosis were officially reported from Algeria, Mauritania, Morocco and Tunisia at the beginning of the last century [24–27]. In-depth epidemiological studies began at the end of the 1980s and showed that camel trypanosomosis could be considered as a dominant disease, with variable prevalence rates depending on the year, the sampling period and the provinces or wilayate (districts) surveyed [28–34]. A recent epidemiological study in southern Algeria carried out on 1056 dromedary camels revealed overall prevalence rates of 2.4% by Giemsa-stained thin smear (GST), 32.4% by card agglutination test for trypanosomosis (CATT/*T. evansi*), 23.1% by enzyme-linked immunosorbent assay (ELISA/VSG RoTat 1.2), 21.0% by immune trypanolysis (TL) and 11.2% by PCR (RoTat 1.2 PCR) [35].

Here, we present a genotyping analysis for six of the camels from the previous study [35], based on sequencing of minicircle DNA and of the F_1_F_O_ ATP synthase subunit γ gene, and confirm the pathogen as *T. b. evansi* type A. Furthermore, we present a novel PCR assay based on primers with improved specificity for minicircle type A that will be useful for typing of surra infections.

## Materials and Methods

All PCR primers are listed in Table 1. All trypanosome isolates or strains are listed in Table 2. *Trypanosoma b. evansi* and *T. b. equiperdum* reference strains were kind gifts from Kirsten Gillingwater, Swiss Tropical Institute [36] and from Philippe Büscher and Nick Van Reet, ITM Antwerp.

**Table 1:** PCR primers used in this study

Primer ID	Target	Sequence	References
1	ATP synthase γ subunit (Tb927.10.180), forward	5′-AACTGCCGTGTCTTGTTGTAA-3′	This study
2	ATP synthase γ subunit (Tb927.10.180), reverse	5′-CGAGTAAGATGGTATTGATGC-3′	This study
3	ATP synthase γ subunit (Tb927.10.180), forward	5′-GCGGAATTCGAAGCAGATGACACCTAA-3′	[1]
4	ATP synthase γ subunit (Tb927.10.180), reverse	5′-GGCGACATTCAACTTCAT-3′	[1]
5	Minicircle type A, forward	5′-CCAACAAACAGAATAACTAATG-3′	This study
6	Minicircle type A, reverse	5′-CTCTCTCACCCTAGTATCTC-3′	This study
7	Maxicircle gene A6, forward	5′-ACGGCGGTTTTGAAAACAC-3′	[37]
8	Maxicircle gene A6, reverse	5′-ATTAACTTATTTGATCTTATTCTATAACTCC-3′	[37]
9	Maxicircle gene ND4, forward	5′-TGTGTGACTACCAGAGAT-3′	[37]
10	Maxicircle gene ND4, reverse	5′-ATCCTATACCCGTGTGTA-3′	[37]
MiniA	Undefined subset of minicircle population, forward	5′-GGGTTTTTTAGGTCCGAG-3′	[17]
MiniB	Undefined subset of minicircle population, reverse	5′-CCGAAAATAGCACGTG-3′	[17]

The underlined nucleotides are not part of the targeted sequence

**Table 2:** Isolates investigated or used in this study

Isolate/strain (notes)	Year/host	Country/region	References
Case 1 (1)	2014/dromedary	Algeria, El Bayadh, Bnoud	[35]
Case 2 (1)	2015/dromedary	Algeria, El Bayadh, Brézina	[35]
Case 3 (1)	2016/dromedary	Algeria, El Bayadh, Brézina	[35]
Case 4 (1)	2015/dromedary	Algeria, Béchar, Abadla	[35]
Case 5 (1)	2015/dromedary	Algeria, Béchar, Mechra HB	[35]
Case 6 (1)	2015/dromedary	Algeria, Béchar, Erg Ferradj	[35]
*T. b. brucei* EATRO 1125 AnTat1.1 90:13 (2)	Laboratory strain	n/a	[38]
*T. b. evansi* CAN86/Brazil (3)	1986/dog	Brazil	[36]
*T. b. evansi* Antat3/3 (2)	1969/capybara	South America	[43]
*T. b. evansi* KETRI 2479 (3)	1980/camel	Kenya, Ngurunit	[17]
*T. b. equiperdum* BoTat1.1 (3)	1924/horse	Morocco	[36]
*T. b. equiperdum* OVI (3)	1977/horse	South Africa	[36]
*T. b. equiperdum* Hamburg (3,4)	unknown/unknown	Unknown	[36]
*T. b. evansi* RoTat1.2 (3)	1982/water buffalo	Indonesia	[36]
*T. b. evansi* Philippines (3)	1996/water buffalo	Philippines	[36]
*T. b. brucei* Lister 427 ' single marker' (2)	Laboratory strain	n/a	[39]
*T. b. equiperdum* American (3,4)	Unknown/horse	USA	[36]
*T. b. equiperdum* AnTat4.1 (3,4)	Unknown/unknown	Unknown	[36]

1, DNA purified from blood put on FTA card; 2, grown *in vitro*; 3, grown in mice; 4, suspected to be *T. (b.) evansi* by Claes *et al.* [3]

### Growth of *T. b. evansi *and *T. b. equiperdum* Reference Strains and DNA Isolation

Trypanosome reference strains were grown in MFI mice and purified from blood using DEAE cellulose as described [40]. DNA extraction was performed using the QIAamp 250 mini blood kit (Qiagen, Hilden, Germany) according to the manufacturer’s instructions.

### Preparation of FTA Punches

The Harris Uni-Core punch tool (Merck, Darmstadt, Germany) and cutting mat were prepared by soaking in 2% (w/v) sodium hypochlorite solution for 10 min, followed by three washes with ddH_2_O, soaking in 70% (v/v) ethanol for 5 min and air drying. Punches from FTA cards were washed three times with 200 µl FTA Purification Reagent (GE Healthcare) for 5 min each and twice with 200 µl TE buffer (10 mM Tris–HCl, 0.1 mM EDTA, pH 8.0) for 5 min each. Punches were dried at 50 °C for 15 min and added directly to PCR reaction tubes.

### PCR Assays

All PCR assays were performed in 25 µl volumes and used FTA card punches or trypanosome genomic DNA (1–5 ng) as indicated (negative controls included additional H_2_O instead). Assays for all targets, with exception of the full-length F_1_F_O_ ATP synthase subunit γ (Tb927.10.180), used the following reagents:

**Table:** 

Reagent	Volume
5 × GoTaq PCR buffer (Promega)	5 µl
MgCl_2_ (25 mM)	2 µl
dNTPs (10 mM)	0.5 µl
GoTaq G2 Hot Start (Promega)	0.125 µl

Specific primers, their volumes, and PCR cycling conditions were as follows:

**Table:** 

Target	Primers (10 µM)	Volume (µl)	Cycling conditions
F_1_F_O_ ATP synthase subunit γ (Tb927.10.180), 511-bp fragment	#1, #2	1	95 °C 5 min 35x (95 °C 30 s, 55 °C 30 s, 72 °C 1 min) 72 °C 10 min
Duplex assay minicircle type A (novel)/F_1_F_O_ ATP synthase subunit γ 511-bp fragment	#3, #4, #5, #6	1.25	95 °C 5 min 40 × (95 °C 30 s, 51 °C 30 s, 72 °C 1 min) 72 °C 10 min
Minicircle type A (novel)	#5, #6	2.5	95 °C 5 min 40 × (95 °C 30 s, 51 °C 30 s, 72 °C 1 min) 72 °C 10 min
Minicircle type A (ref [17])	MiniA, MiniB	2.5	95 °C 5 min 40 × (95 °C 30 s, 51 °C 30 s, 72 °C 1 min) 72 °C 10 min
Maxicircle gene A6	#7, #8	1	95 °C 5 min 35 × (95 °C 30 s, 55 °C 30 s, 72 °C 1 min) 72 °C 10 min
Maxicircle gene ND4	#9, #10	1	95 °C 5 min 40 × (95 °C 30 s, 54 °C 30 s, 72 °C 1 min) 72 °C 10 min

PCR reagents for the full-length F_1_F_O_ ATP synthase subunit γ (Tb927.10.180) gene, including flanking regions, were as follows (25 µl total):

**Table:** 

Reagent	Volume
5 × Phusion PCR buffer (New England Biolabs)	5 µl
Primers #3 and #4 (10 µM)	1.25 µl
dNTPs (10 mM)	0.5 µl
Hot Start Phusion (New England Biolabs)	0.25 µl

PCR cycling conditions for the full-length gene were as follows: 98 °C 30 s, 40 cycles (98 °C 10 s, 60 °C 30 s, 72 °C 1 min), 72 °C 10 min.

### Cloning and Sequencing

All PCR products were cleaned up using the PCR Clean-Up kit from Macherey–Nagel (Dueren, Germany). Sequencing was either direct, using the same primers that had been used for the PCR reaction, or after cloning into pCR-Blunt (Invitrogen; for Phusion PCR products) or into pGEM-T easy (Promega; for GoTaq PCR products), following the manufacturer’s instructions. Cloned products were sequenced using Sanger technology (Edinburgh Genomics or MRC Sequencing Service, Dundee) and standard M13 forward and reverse primers.

### Phylogenetic Analysis

A phylogenetic tree was constructed with IQ-TREE [41], using a maximum likelihood model with HKY + G substitution.

## Results and Discussion

### PCR assays for *Tb*ATPase subunit γ confirm infection with *T. b. evansi* type A

**Fig. 1:**
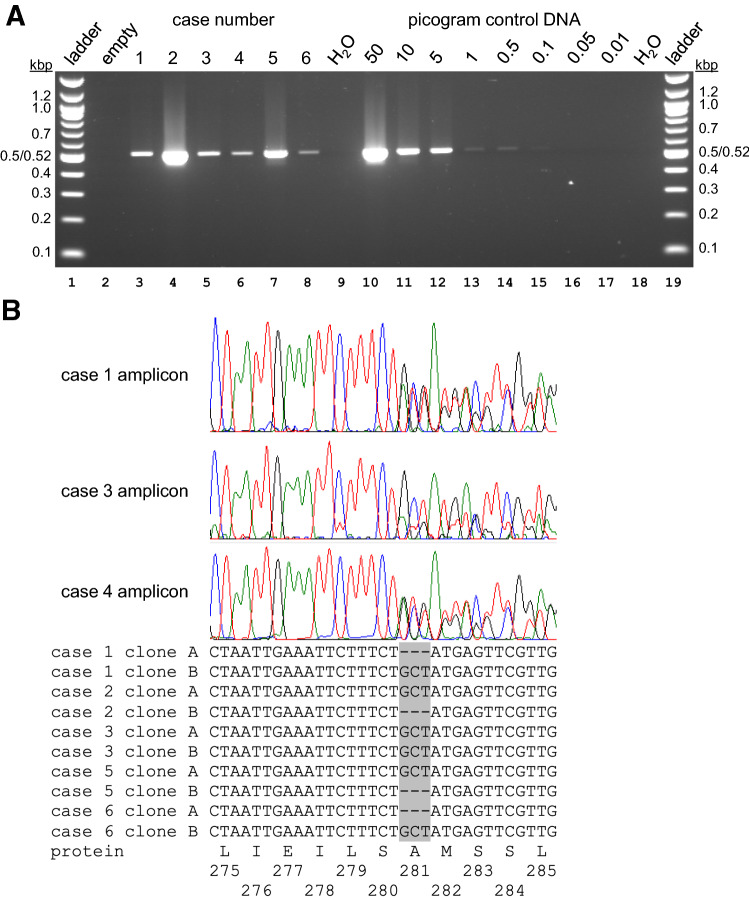
Detection by PCR of ATP synthase subunit γ sequences diagnostic of *T. b. evansi* type A. **A** PCR assay for detection of a 511-bp fragment of ATP synthase subunit γ (Tb927.10.180). Aliquots of completed PCR reactions (15 μl) were fractionated by electrophoresis on an agarose gel containing ethidium bromide. Images were captured using a UV light box. Lanes 1, 19: New England Biolabs 100-bp ladder (kbp: kilobasepairs); lanes 3–8: Algerian camel cases 1–6; lanes 9, 18: PCR reactions with water instead of samples; lanes 10–17: varying amounts of total cellular DNA from *T. b. brucei* strain EATRO 1125 AnTat1.1 90:13. **B** Sequencing of ATP synthase γ sequences. Top, trace files of direct sequencing (from the 5′ end) of PCR amplicons from cases 1, 3 and 4. Bottom, representative sequences obtained after cloning of PCR amplicons. Sequencing of cloned amplicons confirmed that *T. b. evansi* strains responsible for infections 1, 2, 5 and 6 are heterozygous for deletion of amino acid alanine 281 (A281del). All cloned sequences obtained for case 3 were wild-type, and no cloned sequences were obtained for case 4, but direct sequencing of PCR amplicons confirmed heterozygosity for A281del for those cases as well

To confirm the diagnosis of a *T. b. evansi* infection in camels from 5 different Algerian regions (Table 2) [35], we amplified by PCR a 511 bp fragment of subunit γ of the mitochondrial F_1_F_O_ ATP synthase (systematic TriTrypDB ID Tb927.10.180). In the *T. b. evansi* types identified so far, this gene contains adaptive mutations that are differentially diagnostic for types A and B [1, 4, 21]. Punches from FTA cards containing DNA purified from blood samples from cases 1 to 6 were washed and placed in reaction tubes, together with PCR reagents and primers #1 and #2 (Table 1). Initial reactions were carried out with (non-proof-reading) Taq polymerase because of its robust performance. Total cellular DNA from a *T. b. brucei* strain served as positive control. Reactions for all six cases showed a single amplicon of the expected size, suggesting infection with a *Trypanozoon* (Fig. 1A). To identify the type of *T. b. evansi*, we next amplified the entire ATP synthase γ gene with primers #3 and #4 and a proof-reading polymerase, followed by cloning and sequencing. Sequence analysis confirmed presence of a heterozygous A281del mutation in the ATP synthase γ protein for all cases (Fig. 1B), providing conclusive evidence for infection with *T. b. evansi* type A [1]. These results are consistent with the previously reported RoTat1.2-positive PCR results for these isolates [35]. RoTat1.2 is a VSG gene that, when present, is generally considered as being diagnostic for *T. b. evansi* type A [5]. These results are also consistent with the fact that the only other type of *T. b. evansi* currently known, type B, has so far only been reported from countries in East Africa, namely Kenya and Ethiopia [5, 17, 21].

### Development of a Novel PCR Assay Specific for Minicircle Type A

**Fig. 2:**
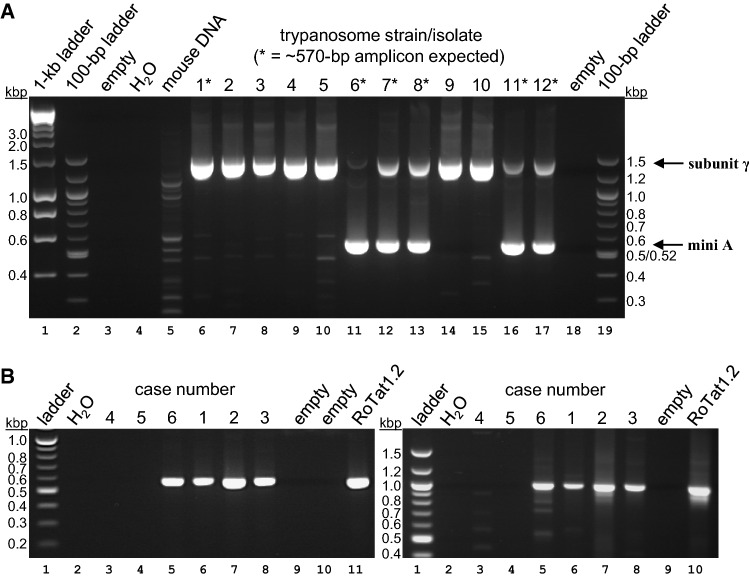
A specific PCR assay for minicircle type A. **A** PCR assay for detection of a ~ 570 bp fragment of minicircle type A (‘mini A’) in samples. In the same reactions (duplex PCR), primers #3 and #4 for amplification of a ~ 1.4-kb ATP synthase subunit γ amplicon (‘subunit γ’) were included as positive internal controls. Per reaction, 1–5 ng total DNA were used as template. Lane 1: Bioline 1-kbp ladder; lanes 2, 19: New England Biolabs 100-bp ladder; lanes 3, 18: empty; lane 4: control PCR reaction with water instead of total DNA; lane 5: control PCR reaction with mouse genomic DNA instead of total trypanosome DNA (several trypanosome strains/isolates were grown in mice); lanes 6–17: reactions with total trypanosome DNA. Trypanosome strains/isolates were as follows. 1 = *T. b. evansi* CAN86/Brazil; 2 = *T. b. evansi* Antat3/3 (akinetoplastic); 3 = *T. b. evansi* KETRI 2479; 4 = *T. b. equiperdum* BoTat1.1; 5 = *T. b. equiperdum* OVI; 6 = *T. b. equiperdum* Hamburg; 7 = *T. b. evansi* RoTat1.2; 8 = *T. b. evansi* Philippines; 9 = *T. b. brucei* Lister 427; 10 = *T. b. brucei* EATRO 1125 AnTat1.1; 11 = *T. b. equiperdum* American; 12 = *T. b. equiperdum* AnTat4.1. Strains/isolates previously identified as belonging to the type A group [1, 3] are indicated by an asterisk. Please note: (i) *T. b. equiperdum* in this group have been suggested to be misidentified or mislabelled *T. b. evansi* [3]; (ii) *T. b. evansi* AnTat3/3 (lane 7) is a type A strain [43], but the strain in our lab had spontaneously lost its kDNA [44]; (iii) *T. b. evansi* CAN86/Brazil is a type A strain [1, 3], but, like AnTat3/3, may have spontaneously lost its kDNA; (iv) amplification of minicircle type A in the same reaction appears to diminish the signal for subunit γ, perhaps by competing for nucleotides, this is particularly evident in lane 11. **B** Analysis of cases 1–6 using the PCR assay with primers #5/#6 (left panel) or primers MiniA/MiniB (right panel). Lane 1: New England Biolabs 100-bp ladder; lane 2: control PCR reaction with water instead of total DNA; lanes 3–8: FTA card punches from cases 1 to 6; lane 9: empty; lane 10: empty (left panel); lane 11 (left panel) / lane 10 (right panel): *T. b. evansi* RoTat1.2 (positive control)

A defining characteristic of *T. b. evansi* type A is that (unless the strain is akinetoplastic [1]) its kDNA is dominated by, or consists entirely of, thousands of copies of a particular class of minicircle [13]. A PCR assay for this minicircle class developed by Njiru and colleagues [17] uses primers (‘MiniA’ and ‘MiniB’) derived from a region semi-conserved among all minicircle classes (Supplementary Figure S1) and can therefore result in false positive reactions [21]. We therefore aimed to develop a PCR assay that is highly specific for type A minicircles. Alignment of type A minicircles available in GenBank identified several regions of perfect conservation outside of the universally conserved region. Based on this information, we designed primer sequences that are predicted to amplify a fragment of ~ 570 bp in a PCR assay (Supplementary Figure S1, primers #5 and #6). Alignment of the minicircle type A consensus to the closest match in the recently defined minicircle population of *T. b. brucei* EATRO 1125 [15], a minicircle that contains the same set of gRNA genes, suggests that primers #5 and #6 should be specific for *T. b. evansi* minicircle type A (Supplementary Figure S2). Indeed, when tested against a panel of type A and non-type A strains or isolates, the PCR assay was highly specific (Fig. 2A). Sequencing of the ~ 570 bp amplicons confirmed that they corresponded to the expected minicircle type A. The only unexpected result was absence of a ~ 570 bp amplicon for strain *T. b. evansi* CAN86/Brazil (Fig. 2A, lane 6). As a PCR reaction using the MiniA/MiniB primers also failed to produce an amplicon for this strain (data not shown), we suspect that this strain has spontaneously lost its kDNA. This phenomenon is not unusual in *T. b. evansi* and *T. b. equiperdum* [14, 42].

Next, we used the new PCR assay to analyze samples from cases 1 to 6. For cases 1, 2, 3 and 6, we obtained a specific band of ~ 570 bp (Fig. 2B, left panel), and direct sequencing confirmed that the amplicons were the type A fragment (Supplementary Figure S3). We did not obtain a product for cases 4 and 5. The same result was obtained with primers MiniA/MiniB: strong amplification products of the expected size for cases 1, 2, 3 and 6, but no products for cases 4 and 5 (Fig. 2B, right panel). We conclude that, in all six cases, camels had been infected with *T. b. evansi* type A. In cases 4 and 5, the parasites may have become akinetoplastic, and our typing relies exclusively on the presence of the A281del mutation of ATP synthase subunit γ. A phylogenetic tree based on the ~ 570 bp minicircle type A amplicon shows a separation into two main branches that is supported by strong bootstrap values (Supplementary Fig. 4). The Algerian cases branch together with two other isolates from Africa, and also a single isolate from South America, whereas the other isolates, all from non-African countries, form a separate branch. It will be interesting to expand the phylogenetic analysis of type A *T. b. evansi* based on their minicircle sequence to include other isolates, perhaps using the entire ~ 1-kb sequence to further improve resolution and reliability.

PCR assays for maxicircle-encoded genes A6 and ND4 (primer pairs #7/#8 and #9/#10, respectively; Table 2) were negative (data not shown), consistent with the expected absence of the maxicircle in *T. b. evansi* [13, 14].

## Conclusion

Based on nuclear and mitochondrial genetic markers, we have confirmed that the recently reported trypanosome infections in southern Algerian camels were caused by *T. b. evansi* type A, adding to an accumulating body of recent reports of surra infections in that country [45–47]. We also report a novel PCR assay based on careful sequence analysis of type A minicircles that we expect will be a useful tool for the community to diagnose *T. b. evansi* type A infections in livestock. Our data reported here suggest good specificity and sensitivity for type A strains and compatibility with samples prepared on FTA cards. Further studies should compare specificity and sensitivity with other assays, such as the recently reported recombinase polymerase amplification lateral flow assay for *T. b. evansi* [48].

## Supplementary Information

Below is the link to the electronic supplementary material.

Supplementary file1 (PDF 293 KB)
