# Supplementary material for: Molecular Analysis of Trypanosome Infections in Algerian Camels
Source: Acta Parasitol. 2022 Jun 3;67(3):1246–53. doi: 10.1007/s11686-022-00577-7 (PMC9399045; doi:10.1007/s11686-022-00577-7)
Supplement: Supplementary file 1 — Supplementary file1 (PDF 293 KB) [file 11686_2022_577_MOESM1_ESM.pdf]

Supplementary Information. Article title: Molecular analysis of trypanosome infections in Algerian camels; Journal: *Acta Parasitologica*
Authors: Boushaki D, Wallis J, Van den Broeck F, Schnaufer A; Corresponding author affiliation and email: Institute of Immunology and Infection Research, University of Edinburgh, achim.schnaufer@ed.ac.uk

```
            1                                                                                               97
            |                                                                                                |
M57459.1    GGGCG-TGCAAATTT-CACCATACACAAAAC-ACGTGCTATTTTCGGGGGTTTTTTAGGTCCGAGGTACTTCGAGAGGGGTTGGTGTAATACACACACGG
M57462.1    GGGCG-TGCAAATTT-CACCATACACAAAAC-ACGTGCTATTTTCGGGGGTTTTTTAGGTCCGAGGTACTTCGAGAGGGGTTGGTGTAATACACACACGG
AY918061.1  GGGCG-gGCAgATTT-CACCATACACAAAtCaACGTGCTATTTTCGGGGGTTTTTgAGGTCCGAGGTACTTCGAaAGGGGTTGGTGTAATACACACACGG
M57460.1    GGGCG-TGCAAATTT-CACCATACACAAAAC-ACGTGCTATTTTCGGGGGTTTTTTAGGTCCGAGGTACTTCGAGAGGGGTTGGTGTAATACACACACGG
M34848.1    GGGCGgTGCAAATTTtCACCATACACAAA-C-ACGTGCTATTTTCGGGGG-TTTTTAGGTCCGAGGTACTTCGAGAGGGGTTGGTGTAATACACACACGG
M81594.1    GGGCG-TGCAgATTT-CACCATACACAAAAC-ACGTGCTATTTTCGGGGGTTTTTTAGGTCCGAGGTACTTCGAaAGGGGTTGGTGTAATACACACAaGG
EU155057.1  GGGCG-TGCAgATTT-CACCATACACAAAAC-ACGTGCTATTTTCGGGGGTTTTTTAGGTCCGAGGTACTTCGAaAGGGGTTGGTGTAATACACACAaGG
M57461.1    GGGCG-TGCAAATTT-CACCATACACAAAAC-ACGTGCTATTTTCGGGGGTTTTTTAGGTCCGAGGTACTTCGAGAGGGGTTGGTGTAATACACACACGG
consensus   GGGCG-TGCAAATTT-CACCATACACAAAAC-ACGTGCTATTTTCGGGGGTTTTTTAGGTCCGAGGTACTTCGAGAGGGGTTGGTGTAATACACACACGG
            *****  *** **** ************* * ***************** **** ****************** ******************** **
              CSB-1                         CSB-2        5'-GGGTTTTTTAGGCCGAG           CSB-3 (UMS)
                                          G-TGCACGATAAAAGCC-5'    MiniA
                                                 MiniB

            98                                                                                             196
            |                                                                                                |
M57459.1    TTTTTCTCAGGGTTTT-GAGaCAATTCGCtaTTTTCCTGgGGTTCTCAGTACACTTAATTTGGATTTAATTGATTTCCTATAGAGaAAAAATAGAATAAT
M57462.1    TTTTTCTCAGGGTTTT-GAGGCAATTCGCAGTTTTCCTGAGGTTCTCAGTACACTTAATTTGGATTTAATTGATTTCCTATAGAG-AAAAATAGAATAAT
AY918061.1  TTTTTCcagGGGTTTT-GAGaCAATTCGCAGTTTTCCTGAGGTTCTCAGTACACTTAATTTGGATTTAATTGAgTTCCTATAGAG-AAAAATAGAATAAT
M57460.1    TTTTTCTCAGGGTTTT-GAGGCAATTCGCtaTTTTCCTGAGGTTCTCAGTACACTTAATTTGGATTTAATTGA-TTCCTATAGAGaAAAAATAGAATAAT
M34848.1    TTTTTCTCAGGGTTTTtGAGGCAATTCGCAGTTTTCCTGAGGTTCTCAGTACACTTAATTTGGATTTAATTGATT--CTATAGAG-AAAAATAGAATAAT
M81594.1    TTTTTCTCAGGGTTTT-GAGGCAATTCGCAGTTTTCCTGgGGTTCTCAGTgCACTTAATTTGGATTTAATTGATTTCCTATAGAG-AAAAATAGAATAAT
EU155057.1  TTTTTCTCAGGGTTTT-GAGGCAATTCGCAGTTTTCCTGgGGTTCTCAGTgCACTTAATTTGGATTTAATTGATTTCCTATAGAG-AAAAATAGAATAAT
M57461.1    TTTTTCTCAGGGTTTT-GAGGCAATTCGCAGTTTTCCTGAGGTTCTCAGTACACTTAATTTGGATTTAATTGATTT-CTATAGAG-AAAAATAGAATAAT
consensus   TTTTTCTCAGGGTTTT-GAGGCAATTCGCAGTTTTCCTGAGGTTCTCAGTACACTTAATTTGGATTTAATTGATTTCCTATAGAG-AAAAATAGAATAAT
            ******   ******* *** ********  ******** ********** ********************** *  ******** *************
                                                                                           18-bp forward

            197                                                                                        291
            |                                                                                            |
M57459.1    AGATAAGTAA-----TCATGAATATAGATATATATAATTGTACATATACCAACAAACAGAATAACTAATGCACAGTGATGATAATAGTTAATTAATTATA
M57462.1    AGATAAGTAA-----TCATGAATATAGATATATATAATTGTACATATACCAACAAACAGAATAACTAATGCACAGTGATGATGAcAGTTAATTAATTATA
AY918061.1  AGATAAGTAA-----TCATGAATATAGATATATATAATTGTACATATACCAACAAACAGAATAACTAATGCACAGTGATGATGATAGTTAATTAATTATA
M57460.1    AGATAAGTAA-----TCATGAATATAGATATATATAATTGTACATATACCAACAAACAGAATAACTAATGCACAGTGATGATGATAGTTAATTAATTATA
M34848.1    AGATAAGTAAgtaatTCATGAATATAGATATATATAATTGTACATATACCAACAAACAGAATAACTAATGCACAGTGATGATGATAGTTAATTAATTATA
M81594.1    AGATAAGTAA-----TCATGAATATAGATATATATAATgGTcCATAT-CCAACAAACAGAATAACTAATGtgCAGTGATGATGATAGTcAATTAATTATA
EU155057.1  AGATAAGTAA-----TCATGAATATAGATATATATAATTGTACATATACCAACAAACAGAATAACTAATGCgCAGTGATGATGATAGTTAATTAATTATA
M57461.1    AGATAAGTAA-----TCATGAATATAGATATATATAATTGTACATATACCAACAAACAGAATAACTAATGCACAGTGATGATGATAGTTAATTAATTATA
consensus   AGATAAGTAA-----TCATGAATATAGATATATATAATTGTACATATACCAACAAACAGAATAACTAATGCACAGTGATGATGATAGTTAATTAATTATA
            **********     ********************** ** ***** *********************   *********** *** ***********
            repeat                                             primer #5 (forward)
                                                                   gCOX3(108-142)

            292                                                                                            391
            |                                                                                                |
M57459.1    TATAAAGTTCTAATCTATCTATTATTATATTTAgTTGAGTGACGTGAGAATAAGGTGATATTTCAATCCTgAACAAAAGAAATGGTGTAATAGATAGAAG
M57462.1    TATAAAGTTCTAATCTATCTATTATTATATTTAATTGAGTGAtGTGAGAATAAGGTGATATTTCAATCCTAAACAAAAGAAATGGTGTAATAGATAGAAG
AY918061.1  TATAAAGTTCTAATCTATCTATTATTATATTTAgTTGAGTGgCGTGAGAATAAGGTGATATTTCAATCCTgAgCAAAAGnAGTGGTGTAGTAGATAGgAG
M57460.1    TATAAAGTTCTAATCTATCTATTATTATATTTAATTGAGTGAtGTGAGAATAAGGTGATATTTCAATCCTAAACAAAAGAAATGGTGTAATAGATAGttG
M34848.1    TATAAAGTTCTAATCTATCTATTATTATATTTAATTGAGTGAtGTGAGAATAAGGTGATATTTCAATCCTAAACAAAAGAAATG-TGTAATAGATAGAAG
M81594.1    TATAAAGTTCTAATCTATCTATTATTATATTTAATTGAGTGgCGTGAGAATAAGGTGATATTTCAATCCTAAACAAAAGgAATGGTGTAATAGATAGAAG
EU155057.1  TATAAAGTTCTAATCTATCTATTATTATATTTAATTGAGTGgCGTGAGAATAAGGTGATATTTCAATCCTAAACAAAAGgAATGaTGTAATAGATAGAAG
M57461.1    TATAAAGTTCTAATCTATCTATTATTATATTTAATTGAGTGAtGTGAGAATAAGGTGATATTTCAATCCT-AACAAAAGAAATGGTGTAATAGATAGttG
consensus   TATAAAGTTCTAATCTATCTATTATTATATTTAATTGAGTGACGTGAGAATAAGGTGATATTTCAATCCTAAACAAAAGAAATGGTGTAATAGATAGAAG
            *****************************************  ************************** * ****** **** ***********  *
                                18-bp reverse repeat                                   18-bp forward repeat

            392                                                                                          489
            |                                                                                              |
M57459.1    ATAATGAGAAGTTAATTATAAATATATCATACAAAATAACAATGATCACAGATAA-GAGTGAATATAGATA-GAGAATTTAATTATTATTATTGTGTATA
M57462.1    ATAATGAGAAGTTAATTATAAATATATCATACAAAATAACAATGATC-CAGATAA-GAGTGAATATAGATA-GAGAATT-AATTATTATTATTGTGTATA
AY918061.1  ATAATGAGAAGTTAATTgTgAATATATCATcCAAAATAACAATGATCcCAGATAA-GAGTGAATATAGATAcGgGAATTTAATTATTATTATTGTGaATA
M57460.1    ATAATGAGAAGTTAATTATAAATATATCATACAAAATAACAATGATCACAGATAA-GAGTGAATATAGATA-GAGAATTTAATTATTATTATTGTGTATA
M34848.1    ATAATGAGAAGTTAATTATAAATATATCATACAAAATAACAATGATCACAGATAAtGAGTGAATATAGATA-GAGAATTTAATTATTATTATTGTGTATA
M81594.1    -TAATGAGAAGTTAATTATAAATATATCATACAAAATAACAATGATCACAGATAA-GAGTGAATATAGATA-GAGAATTTAATTATTATTATTGTGTATA
EU155057.1  -TAATGAGAAGTTAATTATAAATATATCATACAAAATAACAATGATCgCAGATAA-GAGTGAATATAGATA-GAGAATTTAATTATTATTATTGTGTATA
M57461.1    ATAATGAGAAGTTAATTATAAATATATCATACAAAATAACAATGATCACAGATAA-GAGTGAATATAGATA-GAGAATTTAATTATTATTATTGTGTATA
consensus   ATAATGAGAAGTTAATTATAAATATATCATACAAAATAACAATGATCACAGATAA-GAGTGAATATAGATA-GAGAATTTAATTATTATTATTGTGTATA
             *************** * ********** *************** ******* *************** * ***** *************** ***
                                                                gCR4(429-473)

            490                                                                                          588
            |                                                                                              |
M57459.1    TTGAATTACATATTTATTATTTTATTTTAGTATATAGGATGCAGAAAtTAGCAGTATAAAATAA-GGATAAAAGAGTTTATAGGTGAAGTTGAAGTGAAT
M57462.1    TTGAATTACATATT-ATTATTTTATTTTAGTATATAGGACGCAGAAAaTAGCAGTATAAAATAA-GGATAAAcGAGTgTATAGGTGAAGTTGAAGTGgAT
AY918061.1  TTGAATTACATATTTATTATTTTATTTTAGTATATAGGACGCAGAAAaTAGCAGTATAAAATAA-GGATAAAAGAGTTTATAGGTGAAGTTGAAGTGAAT
M57460.1    TTGAATTACATATTTATTATTTTATTTTAGTATATAGGACGCAGAAA-TAGCAGTATAAAATAA-GGATAAAAGAGTTTATAGGTGAAGTTGAAGTGAAT
M34848.1    TTGAATTACATATTTATTATTTTATTTTAGTATATAGGACGCAGAAA-TAGCAGTATAAAATAAtGGATAAAAGAGTTTATAGGTGAAGTTGAAGTGAAT
M81594.1    TTGAATTACATATTTATTATTTTATTTTAGTATATAGGACGCAGAAA-TAGCAGTATAAAATAA-GGATAAAAGAGTTTATAGGTGAAGTTGgAGTGAAT
EU155057.1  TTGAATTACATATTTATTATTTTATTTTAGTATATAGGACGCAGAAA-TAGCAGTATAAAATAA-GGATAAAAGAGTTTATAGGTGAAGTTGAAGTGGAT
M57461.1    TTGAATTACATATTTATTATTTTATTTTAGTATATAGGACGCAGAAA-TAGCAGTATAAAATAA-GGATAAAAGAGTTTATAGGTGAAGTTGAAGTGAAT
consensus   TTGAATTACATATTTATTATTTTATTTTAGTATATAGGACGCAGAAA-TAGCAGTATAAAATAA-GGATAAAAGAGTTTATAGGTGAAGTTGAAGTGAAT
            ************* ******************************** **************** ******* **** ************* **** **
                      18-bp reverse repeat
```

```
           589                                                                                                  688
           |                                                                                                    |
M57459.1   CAGcGTCTTTTGAGGGAAGTAAAGTAATATAATAGATAGAAACATAATAATAATTTAATTTGATAGTATATACATATCAACAACGACAAAGAGTCAGTGA
M57462.1   CAGTGTCTTTTGAGGGAAGTAAAGTAATATAATAGATAGAAACATAATAATAATTTAATTTGATAGTATATACATATCAACAACGACAAAGAGTCAGTGA
AY918061.1 CAGcGTCTTTTGAGGGAAGTAAAGTAATATAATAGATAGAAACATAATAATAATTTAATTTGATAGTATATACATATCAACAACGACAAAGAGTCAGTGA
M57460.1   CAGTGTCTTTTGAGGGAAGTAAAGTAATATAATAGATAGAAACATAATAATAATTTAATTTGATAGTATATACATATCAACAACGACAAAGAGTCAGTGA
M34848.1   CAGTGTCTTTTGAGGGAAGTAAAGTAATATAATAGATAGAAACATAATAATAATTTAATTTGATAGTATATACATATCAACAACGACAAAGAGTCAGTGA
M81594.1   CAGTGTCTTTTGAGGGAAGTAAAGTAATATAATAGATAGAAACATAATAATAATTTAgTTTGgTAGTATATACATATCAACAACGACAAAGAGTCAGTGA
EU155057.1 CAGTGTCTTTTGAGGGAAGTAAAGTAATATAATAGATAGAAACATAATAATAATTTAgTTTGgTAGTATATACATATCAACAACGACAAAGAGTCAGTGA
M57461.1   CAGcGTCTTTTGAGGGAAGTAAAGTAATATAATAGATAGAAACATAATAATAATTTAATTTGATAGTATATACATATCAACAACGACAAAGAGTCAGTGA
consensus  CAGTGTCTTTTGAGGGAAGTAAAGTAATATAATAGATAGAAACATAATAATAATTTAATTTGATAGTATATACATATCAACAACGACAAAGAGTCAGTGA
           *** ***************************************************** **** *************************************
                              18-bp forward repeat                                           gND7(531-576)

           689                                                                                                  788
           |                                                                                                    |
M57459.1   AATTAGAGATAAAGTTATTGTAGTTATATAATTAAATTAATCTATCTATTATTTATTTCTTTTATACGAGGAGAGGGAATAAGAGGGAAAATTCATTGGA
M57462.1   AATTAGAGATAAAGTTATTGTAGTTATATAATTAAATTAATCTATCTATTATTTATTTCTTTTATACGAGGAGAGGGAATAAGAGGGAAAATTCATTGGA
AY918061.1 AATTAGAGATAAAGTTATTGTAGTTATATAATTAAATTAATCTATCTATTATTTATTTCTTTTATACGAGGAGAGGGAATAAGAGGGAAAATTCATTGGA
M57460.1   AATTAGAGATAAAGTTATTGTAGTTATATAATTAAATTAATCTATCTATTATTTATTTCTTTTATA-GAGGAGAGGGAATAAGAGGGAAAATTCATT-GA
M34848.1   AATTAGAGATAAAGTTATTGTAGTTATATAATTAAATTAATCTATCTATTATTTATTTtcTTTtataGAGGAGAGGGAATAAGAGGGAAAATTCAT-GGA
M81594.1   A-TTAGAGATAAAGTTATTGTAGTTATATAATTAAATTAATCTATCTATTATTTATTTCTTTTATACGAGGAGAGGGAATAAGAGGGAAAATTCATTGGA
EU155057.1 AATTAGAGATAAAGTTATTGTAGTTATATAATTAAATTAATCTATCTATTATTTATTTCTTTTATACGAGGAGAGGGAATAAGAGGGAAAATTCATTGGA
M57461.1   AATTAGAGATAAAGTTATTGTAGTTATATAATTAAATTAATCTATCTATTATTTATTTCTTTTATA-GAGGAGAGGGAATAAGAGGGAAAATTCATT-GA
consensus  AATTAGAGATAAAGTTATTGTAGTTATATAATTAAATTAATCTATCTATTATTTATTTCTTTTATACGAGGAGAGGGAATAAGAGGGAAAATTCATTGGA
           * *****************************************************  ***     *****************************  **
                                                    18-bp reverse repeat
           789                                                                                                  885
           |                                                                                                    |
M57459.1   GATACTAGGGTGAGAGAGTTAATAGAGTAAT-GTAGTcGGGAGTATGGAGTAGTTATAA-TTATATTGGCGAAAAGG-AAAGaGCTAAAAAGTCGTGTAG
M57462.1   GATACTAGGGTGAGAGAGTTAATAGAGTAATTGTAGTTGGGAaTATGaAGTAGTTATAA-TTATATTGGTGAAAgGttAAAGGGCTAAAAAGTCGTGTAG
AY918061.1 GATACTAGGGTGAGAGAGTTAATAGAGTAATTGTAGTcGGGAGTATGGAGTAGTTATAA-TTATATTGGCGAAAAGG-AAAGaGCTAAAAAGTCGTGTAG
M57460.1   GATACTAGGGTGAGAGAGTTAATAGAGTAAT-GTAGTT-GGAaTATGGAGTAGTTATAA-TTATATTGGTGAAAAGG-AAAGGGCTAAAAAGTCGTGTAG
M34848.1   GATACTAGGGTGAGAGAGTTAATAGAGTAATTGTAGTTGGGAaTATGGAGTAGTTATAA-TTATATTGGTGAAA-GG-AAAGGGCT-AAAAGTCGTGTAG
M81594.1   GATACTAGGGTGAGAGAGTTAATAGAGTAATTGTgGTTGGGAGTATGGAGTAGTTATAA-TTATATTGGTGAAAAGG-AAAGGGgTAAAAAGTCGTGTAG
EU155057.1 GATACTAGGGTGAGAGAGTTAATAGAGTAATTGTgGTTGGGAGTATGGAGTAGTTATAAaTTATATTGGTGAAAAGG-AAAGGGgTAAAAA-TCGTGTAc
M57461.1   GATACTAGGGTGAGAGAGTTAATAGAGTAATgtag-TT-GGAaTATGGAGTAGTTATAA-TTATATTGGTGAAAAGG-AAAGGGCTAAAAAGTCGTGTAG
consensus  GATACTAGGGTGAGAGAGTTAATAGAGTAATTGTAGTTGGGAGTATGGAGTAGTTATAA-TTATATTGGTGAAAAGG-AAAGGGCTAAAAAGTCGTGTAG
           ******************************     *  *** **** *********** ************* *  **** * * **** *******
           primer #6 (reverse)

           886                                                                                                  985
           |                                                                                                    |
M57459.1   TAGAATAGAGGTTGATAGGAATAAGTGATGGAATTTGTAGAAGggGTTGGTAAAAATCTATAAAAATTGTTAAAATTGGCTAAAAATCGGGCTGAAAAAA
M57462.1   TAGAATAGAGGTTGATAGGAATAAGT-ATGGAATTTGTAGAAGTAGTTGGTAAAAATCTATAAAAATTGTTAAAATTGGCTAAAAATCGGGCTGAAAAAA
AY918061.1 TAGAATAGAGGTTGATAGGAATAAGgGcTGGAATTTGTAGAAGTAGTTGGTAAAAATCTATAgAAATcGTTAAAATTGGCTAAAAATCGGGCTGAAAAAA
M57460.1   TAGAATAGAGGTTGATAGGAATAAGTGATGGAATTTGTAGAAGggGTTGGTAAAAATCTATAAAAATTGTTAAAATTGGCTAAAAATCGGGCTGAAAAAA
M34848.1   TAGAATAGAGGTTGATAGGAATAAGTGgTGGAATTTGTAGAAGggGTTGGTAAAAATCTATAAAAATTGTTAAAATTGGCTAAAAATCGGGCTGAAAAAA
M81594.1   TAGAATAGAGGTTGATAGGAATAAGTGATGGAATTTGTgGAAGTAGTTGGTAAAAATCTATAgAAATcGTTAAAATTGGCTAAAAATCGGGCTGAAAAAA
EU155057.1 TAGAATAGAGGTTGATAGGAATAAGTGATGGAATTTGTgGAAGTAGTTGGTAAAAATCTATAgAAATcGTTAAAATTGGCTAAAAATCGGGCTGAAAAAA
M57461.1   TAGAATAGAGGTTGATAGGAATAAGTGATGGAATTTGTAGAAGggGTTGGTAAAAATCTATAAAAATTGTTAAAATTGGCTAAAAATCGGGCTGAAAAAA
consensus  TAGAATAGAGGTTGATAGGAATAAGTGATGGAATTTGTAGAAGTAGTTGGTAAAAATCTATAAAAATTGTTAAAATTGGCTAAAAATCGGGCTGAAAAAA
           *************************   ********** ****  ***************** **** ********************************

           986         998
           |           |
M57459.1   CGGAAAA-TCTTAT
M57462.1   CGGAAAA-TCTTAT
AY918061.1 CGGAAAAaTCTTAT
M57460.1   CGGAAAA-TCTTAT
M34848.1   CGGAAAA-TCTTAT
M81594.1   CGGAAAA-TCTTAT
EU155057.1 CGGAAAA-TCTTAT
M57461.1   CGGAAAA-TCTTAT
consensus  CGGAAAA-TCTTAT
           ******* ******
```

Supplementary Figure S1. Alignment of type A minicircles from GenBank. For this alignment, position 1 has been defined as the first nucleotide of conserved sequence block 1 (CSB-1) (Ray, 1989). Nucleotide numbering is acording to M57459.1. CSB-1, CSB-2 and CSB-3 (also known as universal minicircle sequence, UMS) are indicated in bold and are underlined. Asterisks below the consensus sequence indicate 100% conservation. Nucleotides in individual sequences that deviate from the consensus are set in lower case. PCR primers MiniA and MiniB (Njiru et al, 2006) are shown aligned to their binding sites in the conserved region. The sequences corresponding to the type A-specific PCR primers developed for the present study, primers #5 and #6, are set in bold and italics. Also indicated are the three gRNAs encoded in this minicircle (wavy lines) and the 18-bp inverted repeats that frame the gRNA gene cassettes (bold and double underlined). GenBank definitions for the database entries are as follows: M57459, *Trypanosoma evansi* kinetoplast DNA sequence; M57462, *Trypanosoma evansi* kinetoplast DNA sequence; AY918061, *Trypanosoma evansi* isolate KETRI 2472 kinetoplast minicircle complete sequence; M57460, *Trypanosoma evansi* kinetoplast DNA sequence; M34848, *T. evansi* AnTat 3/3 kinetoplast DNA minicircle; M81594, *Trypanosoma evansi* DNA; EU155057, *Trypanosoma equiperdum* clone 818mA minicircle, complete sequence; M57461, *Trypanosoma evansi* kinetoplast DNA sequence

```
           1                                                                                                  100
           |                                                                                                    |
mO_359     GGGCGTGCAAAAATACACATACACAAATCCCGTGCTATTTTGGCCTGTTTTTTAGGTCCGAGGTACTTCGAAAGGGGTTGGTGTAATACACACACGGTTT
           |||||||||||..|...||||||||||.|.||||||||||||.|...|||||||||||||||||||||||||||.|||||||||||||||||||||||||||
typeA_cons GGGCGTGCAAATTTCACCATACACAAAACACGTGCTATTTTCGGGGGTTTTTTAGGTCCGAGGTACTTCGAGAGGGGTTGGTGTAATACACACACGGTTT
             CSB-1                    CSB-2          5'-GGGTTTTTTAGGTCCGAG           CSB-3 (UMS)
                                     GTGCACGATAAAAGCC-5'    MiniA
                                             MiniB

           101                                                                                              198
           |                                                                                                  |
mO_359     TT-TCACGGAATTTTTGAATCATAAGGGTTTTCAGGGTTTTAGTGGGAGTACTTGATATGATGGATTTAATTTCGATT-ATTAGATTGTTCCAGGGTAGA
           || |||.||   ||||||        ||...|||...|||||..||.| ||.||...||.     |.|||||||.|||| |.|.|||| |.|.|..|...|
typeA_cons TTCTCAGGG---TTTTGA-------GGCAATTCGCAGTTTTCCTGAG-GTTCTCAGTAC-----ACTTAATTTGGATTTAATTGATT-TCCTATAGAGAA

           199                                                                                              296
           |                                                                   gCOX3(103-139)                 |
mO_359     GAATGAAATAATAGATAG--AATATTAATTTAACTTATAATTATACTATATAAATAACAACAAACGAGATGACCAATACACAGTGATGATGGTATATATT
           .|||..||||||||||||.  |||..|.|.|..|..||||..|| |.|.||.|.|||.||||||||...||.||.|||.||||||||||||||.||..||.|
typeA_cons AAATAGAATAATAGATAAGTAATCATGAATATAGATATATATA-ATTGTACATATACCAACAAACAGAATAACTAATGCACAGTGATGATGATAGTTAAT
           18-bp forward repeat                                      primer #5 (forward)
                                                                   gCOX3(108-142)

           297                                                                                              394
           |                                                                                                  |
mO_359     AATGTCTATATAAGATT-TAATACTTATTTATTACAATATTTTCTTTATTAGGAAG-GAGAAGATGACTGATTTGCGGGATCTAATATAAAGAAGTGGGT
           .|..|.||||||||..|| ||||.  |||.|||||..||||||..||.|.|||.| |||||.|.|. ||||.|.......|||...||||||.|||..
typeA_cons TAATTATATATAAAGTTCTAATC--TATCTATTATTATATTTAATTGAGT--GACGTGAGAATAAGG-TGATATTTCAATCCTAAACAAAAGAAATGGTG
                                18-bp reverse repeat                                          18-bp forward

           395                                                                                              491
           |                                                           gCR4(429-469)                        |
mO_359     TAATAGATAGGTAATTAATTATAATTTAAATGTAACAAT--CATATACAATAACAACAATCGCGAGTAAAGATAGATGTAAGTGAGA-AATTTAACTATA
           ||||||||||. |..||||.|.||.||||.|.|||..||  ||||.|.|||||||||..|||.| ||..||||..||....||..||| |||||||.|||
typeA_cons TAATAGATAGA-AGATAATGAGAAGTTAATTATAAATATATCATACAAAATAACAATGATCAC-AGATAAGAGTGAATATAGATAGAGAATTTAATTAT-
            repeat                                                     gCR4(429-473)

           492                                                                                              585
           |                                                                                                  |
mO_359     TATAA----GAATTGTCTATTA-ATATTTATTATCTTATTTTGTTATATAGGATGCTACGAATAACAGTATAAA-TGTGATGTCGAGATTAAGTAGATTG
           |||.|    |.||..|..|||| ||||||||||||.|||||||..|||||||||||.|| |..||||.|||||||||| |..|......|||.|...|||.|..
typeA_cons TATTATTGTGTATATTGAATTACATATTTATTATTTTATTTTAGTATATAGGACGC-AGAAATAGCAGTATAAAATAAGGATAAAAGAGTTTATAGGTGA
                               18-bp reverse repeat
           586                                                                                              677
           |                                                                                                  |
mO_359     TTTTTC-GTGTAAAACCTGCTAGAGAGTG---TAAAATGATATAATAGATAGATAAGAAGTTAATTATATAGTAAAT--ATAGTATATA--TATCAACAA
           ..||.. |||.|..|....||...|||.|   ||||.|.||||||||||||||    ||..||||.|||...|||.|  ||||||||||  |||||||||
typeA_cons AGTTGAAGTGAATCAGTGTCTTTTGAGGGAAGTAAAGTAATATAATAGATAGA----AACATAATAATAATTTAATTTGATAGTATATACATATCAACAA
                                          18-bp forward repeat
           678                                                                                              777
           |      gND7(528-573)                                                                               |
mO_359     CAGTGAAAAGTCAACGAGATTAGAGATAGAATTATAATTATTGTATATGAATTAAATTAATATATTTATTATTATATTTTTTAATGTTTTGGATAGAGTA
           |....||.|||||..||.||||||||||.|.||||..|..||.||||   |||||||||||.|||.||||||| |||||.||     ||.|          |
typeA_cons CGACAAAGAGTCAGTGAAATTAGAGATAAAGTTATTGTAGTTATATA---ATTAAATTAATCTATCTATTATT-TATTTCTT----TTAT---------A
                  gND7(531-576)                                               18-bp reverse repeat
           778                                                                                              871
           |                                                                                                  |
mO_359     AGAGAAGAGTGGAGTAGATAATAAAAATTAAATTTTAAATTTATAGTCAGGAAAAGTGGGAGAAAATAAGTGTAAATAGATAAAGGGA------ATAATT
           .|||.|||| |||.|| |.|...||||||.|  ||..|..|..|||...|..|.|||.. |.|.|.|||.||||..|.|....|.|||      ||||||
typeA_cons CGAGGAGAG-GGAATA-AGAGGGAAAATTCA--TTGGAGATACTAGGGTGAGAGAGTTA-ATAGAGTAATTGTAGTTGGGAGTATGGAGTAGTTATAATT
                                          primer #6 (reverse)
           872                                                                                              966
           |                                                                                                  |
mO_359     AAATTTGTGTATAGAAGTAGAAGTTTGATGGATAAGTTTACGAATAATAGTGATATATCAGACGAAA-----GATATTGTGGTAGGAGTAGGAGAAATTA
           |.|||.|||.|.||.|    |||....|...|...||.|| |.|.|||||.|.|..||..||..||.     ||..||||.|.||.|||.||..|||.|.
typeA_cons ATATTGGTGAAAAGGA----AAGGGCTAAAAAGTCGTGTA-GTAGAATAGAGGTTGATAGGAATAAGTGATGGAATTTGTAGAAGTAGTTGGTAAAAATC

           967                                                1020
           |                                                     |
mO_359     GATAAAATTATGAGAAATTCGGGTAAAAATCGG-CTGAAAAACCG-AAAATCTTAT
           .||||||.| ||..|||.|.||.|||||||||| ||||||||.|| ||||||||||
typeA_cons TATAAAAAT-TGTTAAAATTGGCTAAAAATCGGGCTGAAAAAACGGAAAATCTTAT
                                                              997
```

Supplementary Figure S2. Alignment of the type A minicircle consensus with its closest match from *T. b. brucei* EATRO 1125. The minicircle type A consensus sequence from Supplementary Figure S1 was aligned to minicircle mO_359 from *T. b. brucei* EATRO 1125 Antat1.1 (Cooper et al., 2019) using EMBOSS Matcher (https://www.ebi.ac.uk/Tools/psa/emboss_matcher/). That minicircle is the closest match in the EATRO 1125 Antat1.1 minicircle by BLAST analysis and contains the same set of gRNAs. Overall identity was 65%; areas of homology were mostly restricted to the conserved minicircle region around conserved sequence blocks CSB-1, CSB-2 and CSB-3 (bold and underlined), the three conserved gRNA genes (wavy lines), and the 18-bp inverted repeats (bold and double underlined) that flank the gRNA genes. PCR primers MiniA and MiniB (Njiru et al, 2006) are shown aligned to their binding sites in the conserved region. The sequences corresponding to the type A-specific PCR primers developed for this study, primers #5 and #6, are set in bold and italics.

```
           261                                                                                                360
           |                                                                                                    |
typeA_cons CACAGTGATGATGATAGTTAATTAATTATATATAAAGTTCTAATCTATCTATTATTATATTTAaTTGAGTGaCGTGAGAATAAGGTGATATTTCAATCCTa
case 1     CACAGTGATGATGATAGTTAATTAATTATATATAAAGTTCTAATCTATCTATTATTATATTTAGTTGAGTGGCGTGAGAATAAGGTGATATTTCAATCCTG
case 2     CACAGTGATGATGATAGTTAATTAATTATATATAAAGTTCTAATCTATCTATTATTATATTTAGTTGAGTGGCGTGAGAATAAGGTGATATTTCAATCCTG
case 3     CACAGTGATGATGATAGTTAATTAATTATATATAAAGTTCTAATCTATCTATTATTATATTTAGTTGAGTGGCGTGAGAATAAGGTGATATTTCAATCCTG
case 6     CACAGTGATGATGATAGTTAATTAATTATATATAAAGTTCTAATCTATCTATTATTATATTTAGTTGAGTGGCGTGAGAATAAGGTGATATTTCAATCCTG
           *************************************************************** ******* ****************************

           361                                                                                                460
           |                                                                                                    |
typeA_cons AACAAAAGAATGgTGTAATAGATAGAAGATAATGAGAAGTTAATTATAAATATATCATACAAAATAACAATGATCACAGATAAGAGTGAATATAGATAGA
case 1     AACAAAAGAATGATGTAATAGATAGAAGATAATGAGAAGTTAATTATAAATATATCATACAAAATAACAATGATCACAGATAAGAGTGAATATAGATAGA
case 2     AACAAAAGAATGATGTAATAGATAGAAGATAATGAGAAGTTAATTATAAATATATCATACAAAATAACAATGATCACAGATAAGAGTGAATATAGATAGA
case 3     AACAAAAGAATGATGTAATAGATAGAAGATAATGAGAAGTTAATTATAAATATATCATACAAAATAACAATGATCACAGATAAGAGTGAATATAGATAGA
case 6     AACAAAAGAATGATGTAATAGATAGAAGATAATGAGAAGTTAATTATAAATATATCATACAAAATAACAATGATCACAGATAAGAGTGAATATAGATAGA
           ************ ***************************************************************************************

           461                                                                                                559
           |                                                                                                    |
typeA_cons GAATTTAATTATTATTATTGTGtATATTGAATTACATATTTATTATTTTATTTTAGTATATAGGACGCAGAAA-TAGCAGTATAAAATAAGGATAAAAGAG
case 1     GAATTTAATTATTATTATTGTGAATATTGAATTACATATTTATTATTTTATTTTAGTATATAGGACGCAGAAAATAGCAGTATAAAATAAGGATAAAAGAG
case 2     GAATTTAATTATTATTATTGTGAATATTGAATTACATATTTATTATTTTATTTTAGTATATAGGACGCAGAAAATAGCAGTATAAAATAAGGATAAAAGAG
case 3     GAATTTAATTATTATTATTGTGAATATTGAATTACATATTTATTATTTTATTTTAGTATATAGGACGCAGAAAATAGCAGTATAAAATAAGGATAAAAGAG
case 6     GAATTTAATTATTATTATTGTGAATATTGAATTACATATTTATTATTTTATTTTAGTATATAGGACGCAGAAAATAGCAGTATAAAATAAGGATAAAAGAG
           ********************** ************************************************** ***************************

           560                                                                                                659
           |                                                                                                    |
typeA_cons TTTATAGGTGAAGTTGAAGTGAATCAGtGTCTTTTGAGGGAAGTAAAGTAATATAATAGATAGAAACATAATAATAATTTAATTTGATAGTATATACATAT
case 1     TTTATAGGTGAAGTTGAAGTGAATCAGCGTCTTTTGAGGGAAGTAAAGTAATATAATAGATAGAAACATAATAATAATTTAATTTGATAGTATATACATAT
case 2     TTTATAGGTGAAGTTGAAGTGAATCAGCGTCTTTTGAGGGAAGTAAAGTAATATAATAGATAGAAACATAATAATAATTTAATTTGATAGTATATACATAT
case 3     TTTATAGGTGAAGTTGAAGTGAATCAGCGTCTTTTGAGGGAAGTAAAGTAATATAATAGATAGAAACATAATAATAATTTAATTTGATAGTATATACATAT
case 6     TTTATAGGTGAAGTTGAAGTGAATCAGCGTCTTTTGrGGGAAGTAAAGTAATATAATAGATAGAAACATAATAATAATTTAATTTGATAGTATATACATAT
           *************************** ******** ***************************************************************

           660                                                                                                759
           |                                                                                                    |
typeA_cons CAACAACGACAAAGAGTCAGTGAAATTAGAGATAAAGTTATTGTAGTTATATAATTAAATTAATCTATCTATTATTTATTTCTTTTATACGAGGAGAGGGA
case 1     CAACAACGACAAAGAGTCAGTGAAATTAGAGATAAAGTTATTGTAGTTATATAATTAAATTAATCTATCTATTATTTATTTCTTTTATACGAGGAGAGGGA
case 2     CAACAACGACAAAGAGTCAGTGAAATTAGAGATAAAGTTATTGTAGTTATATAATTAAATTAATCTATCTATTATTTATTTCTTTTATACGAGGAGAGGGA
case 3     CAACAACGACAAAGAGTCAGTGAAATTAGAGATAAAGTTATTGTAGTTATATAATTAAATTAATCTATCTATTATTTATTTCTTTTATACGAGGAGAGGGA
case 6     CAACAACGACAAAGAGTCAGTGAAATTAGAGATAAAGTTATTGTAGTTATATAATTAAATTAAgCTATCTATTATTTATTTCTTTTATACGAGGAGAGGGA
           *************************************************************** *************************************

           760                779
           |                  |
typeA_cons ATAAGAGGGAAAATTCATTG
case 1     ATAAGAGGGAAAATTCATTG
case 2     ATAAGAGGGAAAATTCATTG
case 3     ATAAGAGGGAAAATTCATTG
case 6     ATAAGAGGGAAAATTCATTG
           ********************
```

Supplementary Figure S3. Alignment of the primer #5/#6 amplicons from cases 1, 2, 3 and 6 with the type A consensus. The minicircle type A consensus from Supplementary Figure S1 was aligned to sequences obtained by direct Sanger sequencing of the PCR amplicons from Fig. 2B (left panel). Primer sequences are not shown. Nucleotides that are 100% conserved among all five sequences are indicated by asterisks. Sequences for cases 1, 2 and 3 are identical but differ from the sequence for case 6 in two positions and from the type A consensus in seven positions.

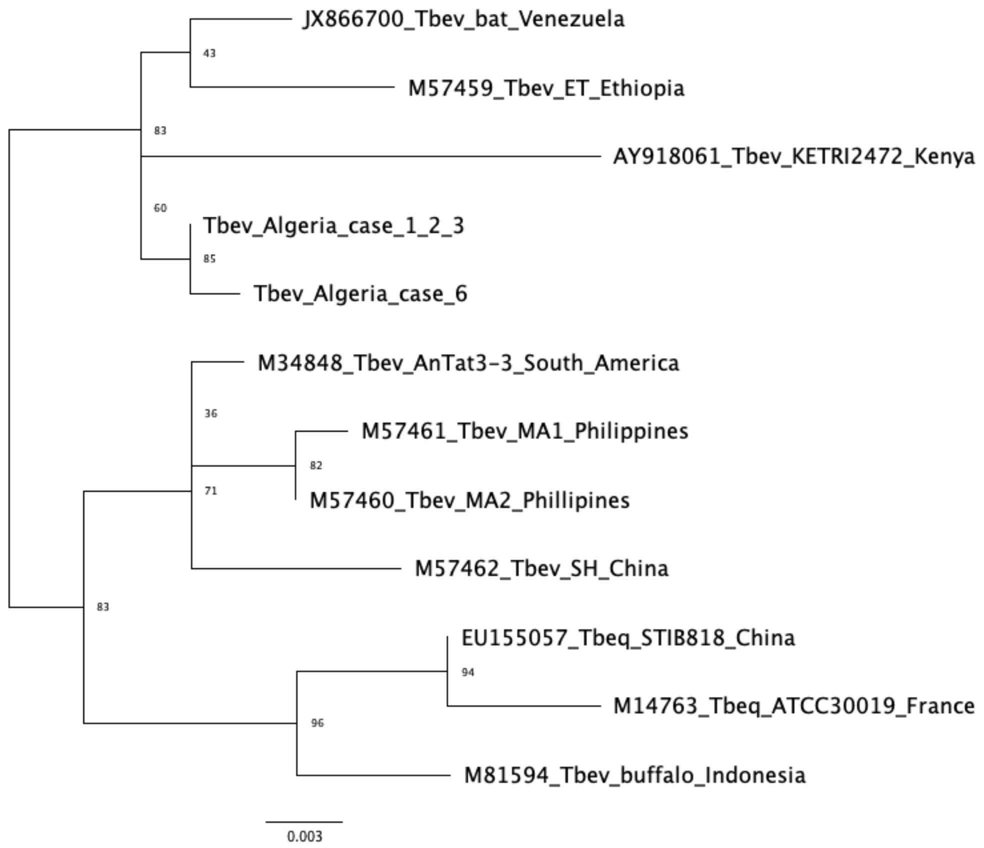

Supplementary Figure 4. Phylogenetic tree for *T. b. evansi* type A based on their minicircle sequences. The ~570-bp amplicons obtained for cases 1-6 were aligned to the corresponding regions from type A minicircle sequences from GenBank (see Supplementary Figures 1 and 3) and a Maximum Likelihood tree with a HKY+G substitution model was generated using IQ-TREE (Nguyen et al., 2015). Node labels indicate bootstrap values; scale bar at the bottom.
